# Meta-analyses triggered by previous (false-)significant findings: problems and solutions

**DOI:** 10.1186/s13643-015-0048-9

**Published:** 2015-04-25

**Authors:** Ewoud Schuit, Kit CB Roes, Ben WJ Mol, Anneke Kwee, Karel GM Moons, Rolf HH Groenwold

**Affiliations:** Julius Center for Health Sciences and Primary Care, University Medical Center Utrecht, Universiteitsweg 100, 3584 CG Utrecht, The Netherlands; Department of Obstetrics and Gynecology, Academic Medical Center, Meibergdreef 9, 1105 AZ Amsterdam, The Netherlands; Stanford Prevention Research Center, Medical School Office Building, Stanford University, 1265 Welch Road, Stanford, CA 94305 USA; The Robinson Institute, School of Reproductive Health and Pediatrics, University of Adelaide, 55 King William Road, North Adelaide, SA 5006 Australia; Department of Obstetrics and Gynecology, University Medical Center Utrecht, Lundlaan 6, 3584 EA Utrecht, The Netherlands

**Keywords:** Meta-analysis, Bias, Type I error, Power

## Abstract

**Background:**

Meta-analyses are typically triggered by a (potentially false-significant) finding in one of the preceding primary studies. We studied consequences of meta-analysis investigating effects when primary studies that triggered such meta-analysis are also included.

**Methods:**

We analytically determined the bias of the treatment effect estimates obtained by meta-analysis, conditional on the number of included primary and false-significant studies. The type I error rate and power of the meta-analysis were assessed using simulations. We applied a method for bias-correction, by subtracting an analytically derived bias from the treatment effect estimated in meta-analysis.

**Results:**

Bias in meta-analytical effects and type I error rates increased when increasing numbers of primary studies with false-significant effects were included. When 20% of the primary studies showed false-significant effects, the bias was 0.33 (*z*-score) instead of 0, and the type I error rate was 23% instead of 5%. After applying a bias-correction, the type I error rate became indeed 5%.

**Conclusions:**

Inclusion of primary studies with false-significant effects leads to biased effect estimates and inflated type I error rates in the meta-analysis, depending on the number of false-significant studies. This bias can be adjusted for.

**Electronic supplementary material:**

The online version of this article (doi:10.1186/s13643-015-0048-9) contains supplementary material, which is available to authorized users.

## Background

Meta-analysis, either using aggregate or individual participant data, involves the synthesis of results or data from several studies and is considered the best design to determine the effects of particular exposures or interventions [1,2]. Meta-analyses do not stand alone; they are often the final analysis of a period of accumulating scientific evidence, either investigating main effects or differential effects of the intervention across subgroups (subgroup effect).

Initially, primary studies are designed to show an overall effect of the treatment on the main or primary outcome. Those studies are often too small (underpowered) to be able to detect subgroup effects or adverse outcomes. Therefore, it makes sense to combine the information obtained from multiple studies, by conducting a meta-analysis, since the statistical power of a meta-analysis will be higher than those of the individual studies. The choice to initiate a meta-analysis may be based on an observed effect or trend towards an effect in a single study. Obviously, such an effect may be falsely significant [3]. As such, these effects, that is, main effects in small trials but also subgroup effects or effects in adverse outcomes for which the primary study was not designed, may in fact be spurious findings [3-6], yet they may trigger the conduct of a meta-analysis. Consequently, meta-analysis including these studies is more prone to bias [7-12].

Previously, Pereira and Ioannidis found that most meta-analyses with significant findings pertain to truly non-null-effects, but that exceptions are not uncommon (that is, more common than the expected 5%) [5]. Their study was based on published meta-analyses, obtained from the Cochrane Database of Systematic Reviews, but did not investigate the consequences for the meta-analysis when the trigger study (potentially false-significant) is included in the meta-analysis and how these consequences (for example, bias in effect estimate of the meta-analysis) could be solved.

Therefore, our aim was to specifically discuss the implication of conducting a meta-analysis that was triggered by results of one or more primary study effects that are also included in that meta-analysis. Throughout the following, we will use the term effect, to indicate a certain treatment effect, which can be either a main effect, subgroup effect, or unintended effect. We stress, however, that a trigger for a meta-analysis will often be a significant finding of a subgroup effect in one of the included studies, rather than the main effect. First, we will derive analytically what the impact is of the inclusion of primary studies with false-significant results in a meta-analysis on the bias in the effect estimate from the meta-analysis. Then, using simulations, we will assess the impact of this conduct on the type I error rate (that is, rate of false-significant studies) and the power (that is, rate of true-significant studies) of meta-analysis. Moreover, we suggest a simple correction method to adjust for potentially inflated type I error rates and bias, using the analytical bias under a null-effect.

## Methods

### Bias in meta-analysis

#### Meta-analysis

In meta-analysis, effect estimates from individual studies are pooled. These estimates can relate to main effects, subgroup effects, effects within subgroups, or unintended effects. As indicated above, throughout this paper, we will use the term effect, to indicate a certain treatment effect, which can be either of these effects.

Suppose that five trials have been conducted in which effects of a certain treatment were assessed. Then, under the assumption of a null-effect, any non-significant study included in the meta-analysis is correctly non-significant, whereas significant ones are false-significant. By chance, one of the five trials may show a false-significant association between treatment and outcome. Since the results of the five trials are inconsistent, this finding may lead to the conduct of a meta-analysis in order to pool all available information to obtain a final answer to the question about the effects of the treatment.

The overall estimate obtained in a meta-analysis usually is a weighted average of the effect estimates of the individual studies, both significant and non-significant, included in the meta-analysis. An overall effect estimate can be calculated by: [13]

1 $$ \mathrm{effect}=\frac{{\displaystyle {\sum}_i{w}_i{\widehat{E}}_i}}{{\displaystyle {\sum}_i{w}_i}} $$

With *w*_*i*_ as the weight that represents the contribution of study *i*, which is equal to the inverse of the within study variance (for a fixed effects model), or equal to the inverse of the within and between study variance τ^2^ (for a random effects model), and *Ê*_*i*_ as the effect estimate in study *i*.

If studies included in a meta-analysis have approximately the same size and overall the treatment has no effect, the combined effect of the non-significant studies will be relatively close to zero, since those studies did not show a significant effect. In contrast, the combined effect of the false-significant (under the assumption of a null-effect) studies will be relatively far from zero. Suppose, a treatment under study has no effect on the outcome, yet a meta-analysis was triggered by a false-significant result in one study while there were five studies included in total. In that case, the overall estimate in the meta-analysis is a weighted average of an effect that is far from zero (one false-significant study) and four effects that are close to zero (four non-significant studies): the estimate in the meta-analysis then likely differs from zero. Obviously, the deviation from zero depends on both the effect estimate in each primary study and the sample size of those studies. Since the true treatment effect is zero, the estimate of the meta-analysis is a biased estimate of the true treatment effect. On average, in the case of endless repetition of meta-analyses (some including significant studies, others not), one still expects to observe a treatment effect of zero, that is, no bias.

#### Analytical derivation of bias

In order to analytically determine the magnitude of the bias of the treatment effect obtained in the meta-analysis, we consider a continuous outcome and the treatment effect is defined as the difference of the mean outcome value of the two treatment groups (A and B). In the following, we assume that the true treatment effect equals zero.

**Figure 1:**
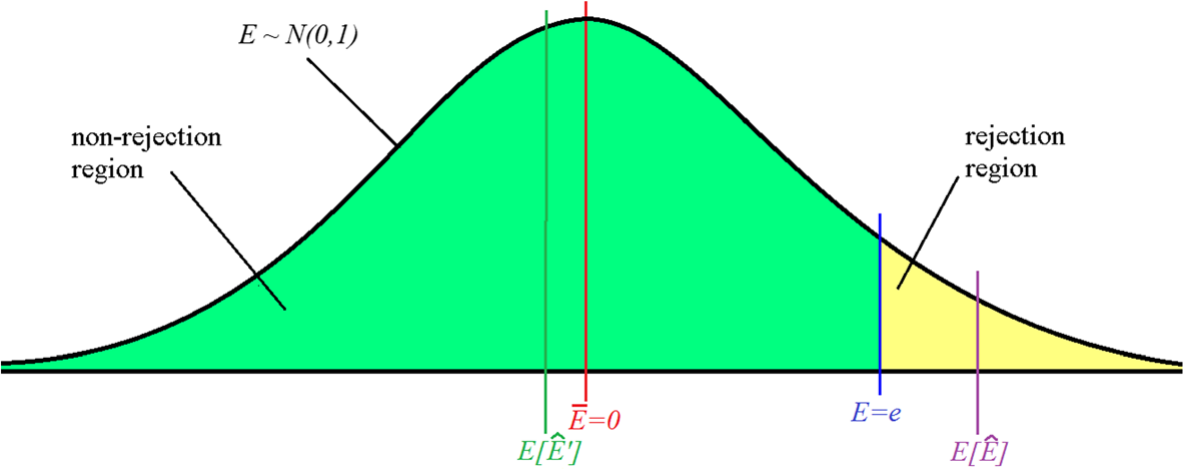
Truncated standard normal distribution. The overall mean of *E*, *Ē*, is zero. Truncation at *E = e* splits up the distribution into a rejection region and non-rejection region. These regions have an expected mean of *E*[*Ê*] and *E*[*Ê* ^’^], respectively [14]. For *e* = 1.65, *E*[*Ê* ^'^] = −0.108 and *E*[*Ê*] = 2.07.

The amount of bias in the effect estimate in the meta-analysis is defined as the average difference between the estimated effect in the meta-analysis (Equation 1) and the true treatment effect. In the case of no effect, the mean effect of a meta-analysis is expected to be zero, and the test-statistic of the effect follows a *z*-distribution. The effect estimate obtained from a meta-analysis is a weighted average of the effects from the included studies, which include both significant and non-significant studies. In the significant studies, the effect should exceed a certain value, in order to achieve significance. Similarly, in the non-significant studies, the effect will not exceed this value. Based on a known boundary of significance (for example, 0.05 significance level), and under the assumption of a null-effect, the expected means of the significant and non-significant studies can be derived from a truncated normal distribution (Figure 1) [14]. In the case of a standard normal distribution and a one-sided test at a 0.05 significance level, the significant effects will lie in the rejection region of a normal distribution that is truncated at a *z*-score of 1.65, whereas the non-significant effects will lie in the non-rejection region of that truncated normal distribution. The expected mean effects of the non-significant studies (*E*[*Ê*^’^]) and the significant studies (*E*[*Ê*]) can be calculated as described by Barr and Sherrill [14] and are under a null-effect approximately equal to −0.108 and 2.07, respectively, for a one-sided significance level of 0.05. Suppose that under a null-effect, a meta-analysis was based on the significant results in one study (that is, false-significant) while there are five studies included in total, then the overall effect estimate obtained by meta-analysis is expected to be (4 * (−0.108) + 1 * (2.07)) / 5 = 0.33. Notice that this overall expectation of the effect estimate differs from zero and is thus biased since the true effect was zero. In the case of a two-sided statistical test, two significant studies may have opposite effects which may cancel out. Therefore, the above approach is only applicable to a one-sided test. We do acknowledge that most meta-analyses apply two-sided statistical tests and we come back to this issue in the discussion of this paper.

Apart from the mean effects of the significant and non-significant studies, the size of this bias depends on the number of small and large studies, which are significant or non-significant. Under a null-effect, the effect estimate is equal to the bias and can, under a fixed effects approach, be derived from Equation 1 (Additional file 1):

2 $$ \mathrm{effect}=\frac{\left({n}_{s_s}{N}_s+{n}_{l_s}{N}_l\right)\cdot 2.07+\left({n}_{s_{ns}}{N}_s+{n}_{l_{ns}}{N}_l\right)\cdot -0.108}{n_{s_s}{N}_s+{n}_{l_s}{N}_l+{n}_{s_{ns}}{N}_s+{n}_{l_{ns}}{N}_l} $$

With ${n}_{s_{ns}}$ as the number of non-significant small studies, ${n}_{l_{ns}}$ as the number of non-significant large studies, ${n}_{s_s}$ as the number of significant small studies, ${n}_{l_s}$ as the number of significant large studies, *N*_*s*_ as the sample size of the small studies, *N*_*l*_ as the sample size of the large studies, −1.08 as the expected mean difference between treatments A and B in the non-significant studies, and 2.07 as the expected mean difference between treatments A and B in the significant studies (that is, 2.07 for a one-sided 0.05 significance level).

### Simulation studies of statistical inference in meta-analysis

#### Simulation set-up

We set up a simulation study to assess the performance of statistical analysis in meta-analysis, that is, the type I error rate (that is, rate of incorrect rejection of the null-hypothesis) and power (rate of correct rejection of the null-hypothesis). We considered a continuous outcome, measured in individuals allocated to treatments A and B. To evaluate type I error rates, we simulated a zero effect while we simulated a difference to evaluate power. Under the assumption of a difference in effect of 0.2 between treatments A and B, a variance of 1, a desired power of 80%, and a type I error rate of 5%, approximately 330 individuals were needed in each treatment arm of the meta-analysis. The individuals were divided over 10 studies, with the proportion of small studies in the meta-analysis varying from 10% to 80% and the ratio of the sample sizes in the small and large studies ranging from larger studies that had a sample size that was equal, or 2, 4, or 8 times larger than that of the small studies (Table 1). Note that since the sample size was calculated based on the overall meta-analysis, all individual studies had a power of less than 80%. These sample sizes were used in both scenarios.

**Table 1:** **An overview of all simulated scenarios**

**Number of small studies out of ten total studies**	**Sample size per study per arm for different ratios of small and large studies** ^**a**^					
	**1:8**		**1:4**		**1:2**	
	**Small studies**	**Large studies**	**Small studies**	**Large studies**	**Small studies**	**Large studies**
1	5	40	9	36	17	34
2	5	40	10	40	18	36
3	6	48	11	44	19	38
4	6	48	12	48	21	42
5	7	56	13	52	22	44
6	9	72	15	60	24	48
7	11	88	17	68	25	50
8	14	112	21	82	28	56

All scenarios were simulated 1,000,000 times and were used to assess the type I error rate and the power of the overall meta-analysis using both a fixed and a random effects models. To assess the type I error rate, a zero effect was simulated using a standard normal distribution (mean 0, variance 1) for both treatment arms. To assess the power, a treatment effect of 0.2 was simulated using a standard normal distribution for treatment A and a normal distribution (mean 0.2, variance 1) for treatment B. ^a^All meta-analyses combined roughly 330 individuals per treatment arm (numbers may deviate due to rounding off).

For each scenario, we determined the number of small studies and the ratio in sample size between small and large studies. Then, within each simulation, we simulated continuous outcomes for all individuals included in the meta-analysis (either assigned to treatment A or treatment B). To assess the type I error rates, the continuous outcome of individuals allocated to treatments A or B was drawn from a standard normal distribution (that is, mean 0, variance 1) to simulate a true zero effect. In the second scenario, to simulate a non-zero effect, we simulated a 0.2 difference in the continuous outcome between treatments A and B, by sampling outcomes from a standard normal distribution for treatment A and from a normal distribution with a mean 0.2 and a variance 1 for treatment B. Then, based on the number of small studies and the ratio between the sample size of the small and large studies, the simulated individuals were distributed over the ten individual studies.

The treatment effect (that is, the difference in the mean outcome value between treatments A and B) and its significance (based on a one-sided two-sample *t*-test) were estimated in each individual study. Next, the study level results of the ten studies were combined. and the mean effect estimate and its significance were estimated on the meta-analysis level using a fixed as well as a random effects model. Effect estimates from individual studies were weighted by the inverse of the variance within studies in a fixed effects approach and by the inverse of the variance within and between (τ^2^) studies in the random effects approach [13]. The type I error rate was estimated by the mean number of false-significant meta-analyses (simulated a zero effect), while the power was estimated as the mean number of true-significant meta-analyses (simulated a true effect).

To assess the type I error rate and the power, simulation were performed 1,000,000 times. Such a large number was needed to assess the type I error rate (scenario 1) and power (scenario 2) in a situation that, for example, four studies showed a false-significant result. Say that ten studies are simulated under a null-effect, then the chance that three out of these ten studies are false-significant is 0.05^3^ * 0.95^7^ * 10 = 0.00087. Therefore, to be able to draw conditional conclusions (for example, if three studies showed a false-significant result), such large numbers of simulation runs were needed. The large number of simulations resulted in a standard error of the type I error rate of 0.0002179449 in the case of three false-significant studies in a meta-analysis of ten studies. Hence, averaged type I error rates have a 95% probability of being in the range of 4.96 to 5.04% if the true type I error rate was indeed 5%. The standard error of the power was 0.0004, that is, averaged power has a 95% probability of being in the range of 79.93 to 80.07%, if the true power was indeed 80%.

#### Bias-correction

A possible solution to correct for a potentially inflated type I error rate and inadequate power is to apply a bias-correction based on the analytical bias, which was derived above. To evaluate this approach, we subtracted the analytical bias (calculated using Equation 2) from the effect estimate in the meta-analyses, thereby correcting for the sample size of the individual studies by multiplying the bias by $\frac{4}{N_i}$, where *N*_*i*_ indicates the sample size of a study (either small or large). Next, we determined the type I error rate and power again using this corrected estimate. All statistical analyses were conducted using R for Windows, version 2.15.2 [15].

## Results

### Bias in meta-analysis

**Figure 2:**
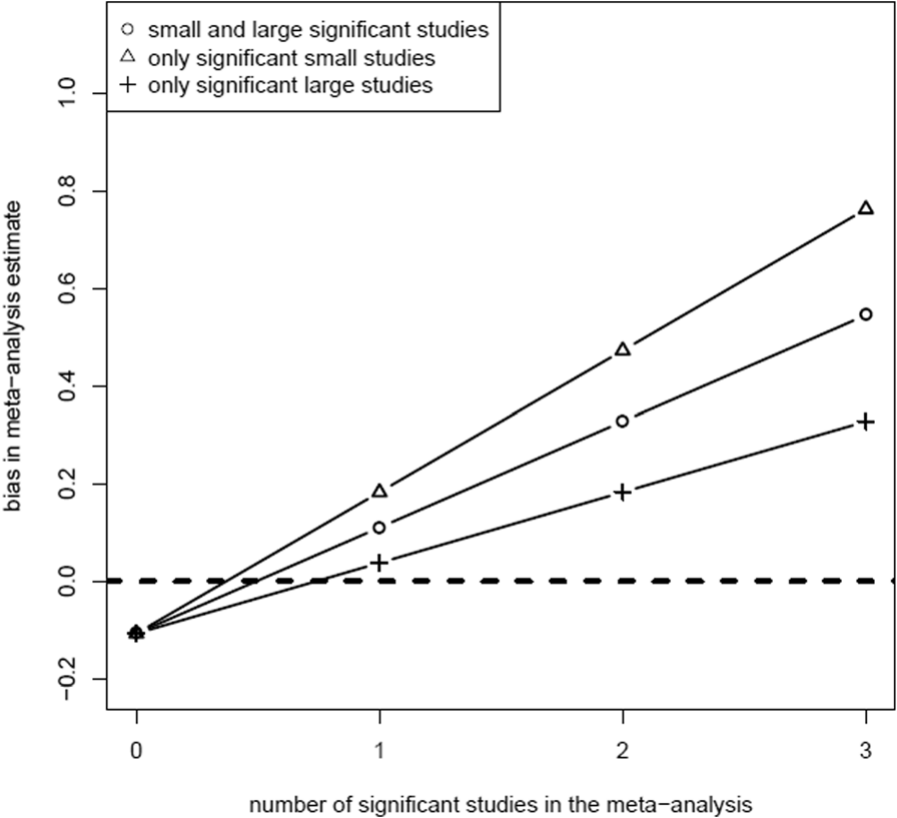
Bias in effect estimates from meta-analysis due to inclusion of false-positive studies. The dashed horizontal line indicates the bias over all simulated meta-analyses, which is equal to zero. The triangles indicate the bias in relation to the number of false-significant studies (we assumed no treatment effect) in the meta-analysis when only large studies showed a false-significant effect. The crosses show the bias when all false-significant studies were small.

Application of Equation 2 (that is, under the assumption of no difference in the mean outcome value between treatments A and B) showed that the bias in the overall effect obtained in the meta-analysis increased with an increasing number of false-significant studies included in the meta-analysis (Figure 2). When none of the total ten studies showed a false-significant result, the bias was negative, while a positive bias was found when one or more studies had a false-significant result. The upper bound of the bias was found when all of the significant studies were large (that is, none of the small studies showed a false-significant effect), while the lower bound of the bias was found when all false-significant studies were small (that is, none of the small studies showed a false-significant effect). For other combinations, for example, one large false-significant study and an increasing number of false-significant small studies, the bias of the effect estimate from the meta-analysis was found to be in-between the extremes (data not shown).

### Simulation studies of statistical inference in meta-analysis

**Figure 3:**
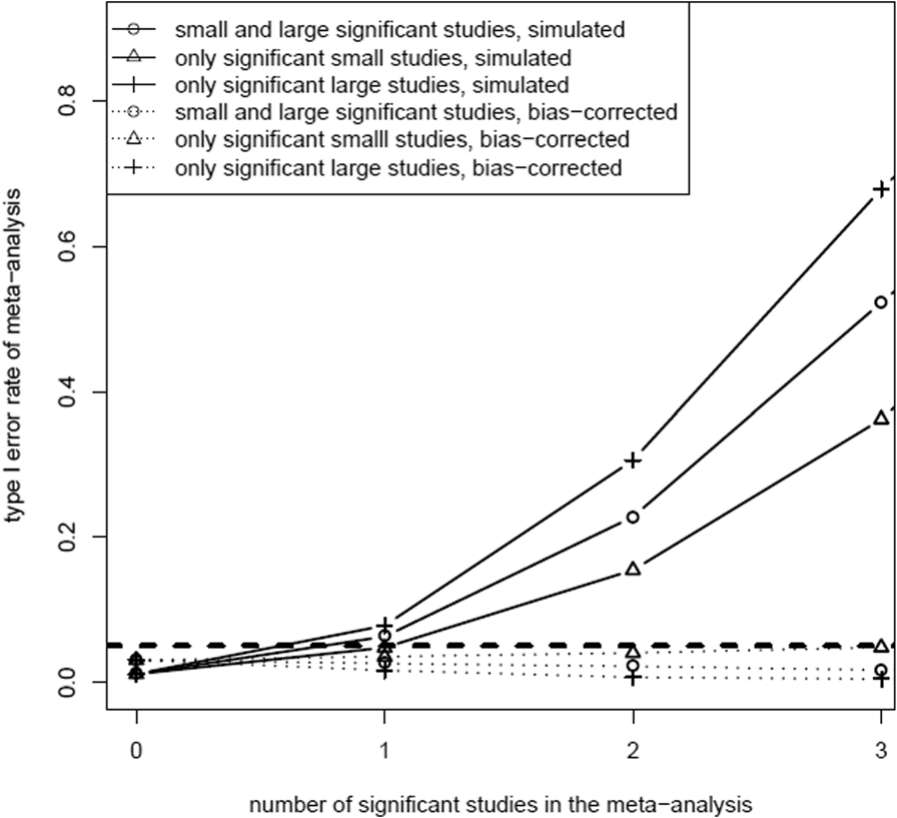
Type I error rate in meta-analysis in relation to the number of false-significant studies (we assumed no treatment effect) included in the meta-analysis. The dashed horizontal line indicates the type I error of all simulated meta-analyses together, which is equal to 5%. The solid lines show the type I error rate obtained by simulation and the dotted lines indicate the type I error rate after bias-correction. The triangles indicate the type I error rate in relation to the number of false-significant studies in the meta-analysis when all false-significant studies were large. The crosses show the type I error rate when all false-significant studies were small.

The results of the simulation study with no difference between treatments A and B and the use of a fixed effects model showed that the type I error rate of the overall meta-analysis increased when the number of studies with a false-significant effect included in the meta-analysis increased (Figure 3). The most extreme type I error rates were found when only large studies were false-significant (highest) or when only small studies included in the meta-analysis showed a false-significant effect (lowest). Other combinations of false-significant small and large studies resulted in a type I error rate of the overall meta-analysis that was in-between these boundaries (data not shown). After the bias-correction, the type I error rate of the meta-analysis was below 5%, independent of the number of studies with a false-significant effect (Figure 3). As expected, the type I error rate was lower when using a random effects model than seen for a fixed effects model but was still substantial (data not shown). When only the small studies were false-significant, the type I error rate for a meta-analysis using a random effects model showed to be similar to the fixed effects model, as expected.

**Figure 4:**
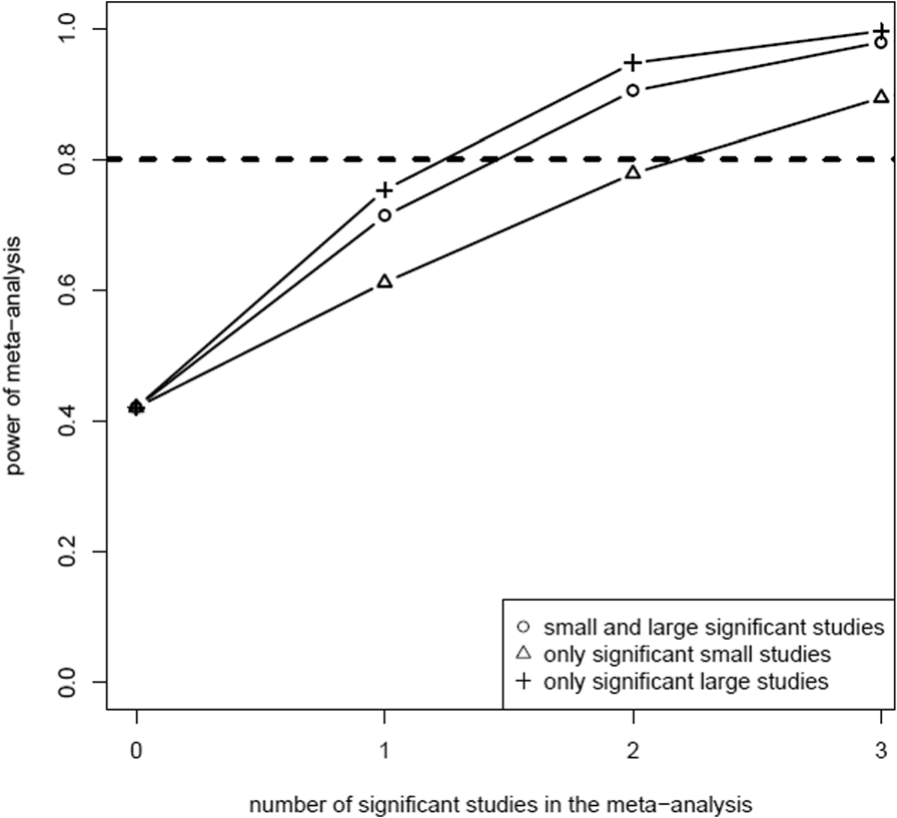
Power in the meta-analysis in relation to the number of true-significant studies (we assumed a treatment effect) included in the meta-analysis. The dashed horizontal line indicates the power of all simulated meta-analyses together, which is equal to 80%. The triangles indicate the power in relation to the number of true-significant studies in the meta-analysis when all true-significant studies were large. The crosses show the power when all true-significant studies were small.

Under a simulated treatment effect of a mean difference of 0.2 between treatments A and B and using a fixed effects model, the power of the meta-analysis increased when the number of studies with a true-significant effect in the meta-analysis increased (Figure 4). When there were no true-significant studies included in the meta-analysis, the power of the meta-analysis was approximately 50%. This increased to about 70% with inclusion of one true-significant study and approached 90% when two or more true-significant studies were included. The extremes of the power of the meta-analysis were found when only large studies were true-significant (highest) or when only small studies included in the meta-analysis showed a true-significant effect (lowest). The bias-correction (that is, subtracting the amount of bias under the null-hypothesis from the effect estimate in the meta-analysis) did not affect the power of the meta-analysis if a non-zero treatment effect was present, since the simulated effect (0.2) was substantially higher than the effect that was corrected for (0). As expected, the random effects model resulted in less power of the meta-analysis as compared to the fixed effects model for all possible combinations of true-significant and false-negative small and large studies (data not shown). The power of the overall meta-analysis was only marginally influenced by the proportion of small studies (varied from 10 to 80% of the total number of included studies) and the ratio of the sample size between the small and large studies (sample size in large studies was 2, 4 or 8 times larger than in the small studies) in the meta-analysis (that is, similar to solid line with circles in Figure 4).

## Discussion

We showed that inclusion of primary studies with false-significant effects in a meta-analysis may bias the effect estimates from that meta-analysis. Consequently, the type I error rates will not meet the prespecified nominal level (usually 5%), particularly when the number of false-significant studies included in a meta-analysis is high and the false-significant studies are among the larger ones included for meta-analysis. Using a simple correction based on the number of included studies, the number of included significant studies, and the sample sizes of the included studies, the nominal type I error rate can be controlled without losing power of the meta-analysis in the event of a non-zero treatment effect.

Our simulations show that applying bias-correction is an effective way to maintain the nominal type I error rate. We determined this bias-correction analytically assuming a *z*-distribution of the ratio of the effect estimate and its standard error of the studies included in the meta-analysis. Consequently, this approach for analytical bias may be representative for other statistics that follow a *z*-distribution or a chi-square distribution, such as the log (odds ratio). Hence, the results of this study are generally applicable, but the particular form of bias-correction may differ.

There are several findings from our study that need further elaboration. First, a negative bias (that is, a bias in the direction opposite to the direction of the one-sided alternative statistical hypothesis) was found for the situation in which none of the included studies showed a false-significant effect (under the assumption of no treatment effect) (Figure 2). This is a result of applying a one-sided test; the average effect size of the meta-analysis is expected to be negative since none of the effects from the included primary studies exceeded a certain positive threshold. If a two-sided test were applied, the average effect size of a meta-analysis in which only true negative studies (that is, correctly non-significant) are included would be zero, because positive as well as negative extreme effect would be excluded. As a consequence, the magnitude of the bias due to selective inclusion into a meta-analysis in the presence of an effect cannot be derived. However, it is to be expected that in the presence of a true positive effect, particular studies with such a positive effect will be significant and thus excluded from the meta-analysis resulting in a negative bias. Likewise, in the presence of a true negative effect, the meta-analysis will be positively biased.

Second, if there were no studies with a true-significant effect included in the meta-analysis (that is, under the assumption of a non-zero treatment effect), the power of the meta-analysis was close to 50% (Figure 4), which is substantially lower than 80%. This is remarkable because the aim of a meta-analysis is often to combine all available primary studies, commonly including a majority of non-significant primary studies, in order to obtain a more precise and indeed possibly significant effect estimate in the meta-analysis. When none of the individual studies included in the meta-analysis shows a (true) significant effect, even though the true effect is for example, 0.2 (as in our example), the pooled effect in the meta-analysis will consequently be lower than 0.2 because of the aforementioned negative bias. As a result, the statistical power to detect this effect will decrease. The distribution of standardized effect sizes (*z*-scores) of adequately sized meta-analyses (adequately sized to have a power of 80% when the type I error rate is set at 5%) will approximately correspond to a normal distribution of mean 2.83 and standard deviation 1. However, the distribution of standardized effect sizes in the selected group of meta-analyses that only include non-significant trials is shifted: mean 1.61 and standard deviation 1. Since the mean of the latter distribution is close to a test-statistic of 1.65 (corresponding to a one-sided significance level of 5%), approximately half of all meta-analyses without any significant studies will have a significant result. Hence, the power of such a meta-analysis is close to 50%.

Third, the power of the meta-analysis could be maintained and was only marginally influenced after applying our bias-correction. In our simulations, the effect estimate of the meta-analysis with an increasing number of true-significant studies will be higher than the simulated true effect size (0.2). Therefore, subtracting the analytical bias (based on a null-hypothesis) will still result in an overall power of 80%.

Fourth, the type I error rates and power were lower when applying a random effects model than using a fixed effects meta-analytical model. This was expected since a random effects model gives, under the simulation of fixed effects, less efficient treatment effect estimates. In a random effects model, the weights of the studies with a higher precision (that is, the studies with larger sample sizes) are lower compared to a fixed effects model. Consequently, the influence of the larger studies on the effect estimates in the meta-analysis is smaller; therefore, the effect estimate of the difference between treatments A and B in the meta-analysis will be lower and less often significant. This results in a lower type I error rate as well as a lower power.

As with every simulation study, assumptions had to be made. In this study, we focused on two scenarios of which one simulated a treatment difference of 0.2 on a continuous scale between both treatment arms and in which the meta-analysis included ten studies. Additionally, in the derivation of Equation 2 (see Additional file 1), we assumed that the variance of the outcome to be the same over all studies, the sample sizes in both arms to be similar over studies, and the sample size and treatment effect were independent. Obviously, results may be different for large (dichotomous) treatment effects, larger or smaller meta-analyses, and unbalanced treatment arms or more diverse in the sample size of the included studies; these topics were beyond the scope of this study.

In this study results, we performed both fixed effects meta-analysis and random effects meta-analysis using the method of DerSimonian and Laird, as these are the two most often used methods in scientific literature [13]. However, especially when the number of studies is small or when substantial differences among study estimates exist, the method of DerSimonian and Laird may produce confidence interval that are too narrow [16]. Therefore, alternative, more reliable methods could be considered including small-sample adjustments, profile likelihood, or hierarchical Bayesian models [16]. However, to illustrate the phenomenon that type I error rates depend on the number of significant studies included in a meta-analysis, we focused on more straightforward methods.

All analyses in this study focused on meta-analyzing a main treatment effect. In general, false-significant main effects are less likely than false-significant secondary, safety, or subgroup effects, because primary studies are in principle designed to reliably investigate a treatment effect on the main outcome. As such, the problem of the initiation of a meta-analysis based on a false-significant study is more prominent in the case of meta-analysis for secondary, safety, or subgroup effects. Therefore, researchers should be especially aware of this problem in such meta-analyses.

## Conclusions

Meta-analyses are typically triggered by an effect in one of the preceding primary studies; a finding that may be false-significant. Inclusion of primary studies with false-significant effects leads to biased effect estimates and inflated type I error rates in the meta-analysis, depending on the number of false-significant studies. The goal in each new study is to replicate previous findings. When true replication of the effect is the aim, a solution to lower the bias and type I error rate would be to exclude the study that triggered the meta-analysis. Alternatively, this bias can be solved by endless repetition of meta-analyses, which we consider impossible and, therefore, the proposed bias-correction, in which the trigger study is included in the meta-analysis, appears to be a viable solution.

## Additional file

Additional file 1:
**Derivation of Equation** 2
