# Supplementary material for: Meta-analyses triggered by previous (false-)significant findings: problems and solutions
Source: Syst Rev. 2015 Apr 25;4:57. doi: 10.1186/s13643-015-0048-9 (PMC4458016; doi:10.1186/s13643-015-0048-9)
Supplement: Additional file 1: — Derivation of Equation 2 [file 13643_2015_48_MOESM1_ESM.doc]

**Additional file 1**

The overall effect observed in a meta-analysis is a weighted average of the effects in the included studies:

(1)

With $w_{i}$ the weights that represent the contribution of each study and are equal to the inverse of the within study variance (fixed effects model) or equal to the inverse of the within and between study variance τ^2^ (random effects model), and $\hat{E}_{i}$ the effect estimate in study *i*.

We consider a continuous outcome under a fixed effects model and given that we can have $n_{s}$ studies with significant and $n_{ns}$ studies with non-significant results, equation (1) can be rewritten as:

(2)

Still assuming a continuous outcome and that the effect can be expressed as a difference in means, we can write $w_{i}$ as:

(3)

With $n_{i}$ the number of subjects per treatment arm within one study and$N_{i}$ the number of subjects within one study. We assume that both treatment arms contain an equal number of subjects, hence *N_i_ = 2n_i_*. Since the weight of each study includes the constant 4 in the denominator and the weights are included in both the numerator and denominator of equation (2), this constant can be dropped and thus the weight of study *i* is given by $\frac{N_{i}}{\mathrm{var}(x)}$.

Suppose that $\mathrm{var}(x)$ is the same for all studies, then equation (2) can be rewritten as:

(4)

If the sample size of a trial and the effect size in a trial are independent then:

(5)

with $E\left[\hat{E}\right][]$ the expectation of the main effect. Now equation 4 can rewritten into:

(6)

Where $n$ denotes the number of small or large (subscript s or l) studies with a significant or non-significant effect (sub-subscript s or ns), $N$ the number of subjects in a small or large study (subscript s or l), and $E\left[\hat{E}\right][]$ denotes the expected effect in the significant studies (i.e. conditional expectation), and $E\left[\hat{E}\right][]$ the expected effect in the non-significant studies.

Following what is described in the *Analytical derivation of bias* section, the conditional expectation among significant studies of , i.e. that under the assumption of a null effect the standardized effect is greater than 1.65, is estimated to be 2.07, and that the conditional expectation among non-significant studies of is estimated to be -0.109. Consequently, equation (6) can be rewritten as:

$effect=\frac{\left(n_{s_{s}}N_{s}+n_{l_{s}}N_{l}\right)\cdot2.07+\left(n_{s_{ns}}N_{s}+n_{l_{ns}}N_{l}\right)\cdot-0.109}{n_{s_{s}}N_{s}+n_{l_{s}}N_{l}+n_{s_{ns}}N_{s}+n_{l_{ns}}N_{l}}$ (7)
